# Transcriptome analysis reveals mechanism underlying the differential intestinal functionality of laying hens in the late phase and peak phase of production

**DOI:** 10.1186/s12864-019-6320-y

**Published:** 2019-12-12

**Authors:** Wei-wei Wang, Jing Wang, Hai-jun Zhang, Shu-geng Wu, Guang-hai Qi

**Affiliations:** Laboratory of Quality & Safety Risk Assessment for Animal Products on Feed Hazards (Beijing) of the Ministry of Agriculture & Rural Affairs, National Engineering Research Center of Biological Feed, Feed Research Institute, Chinese Academy of Agricultural Sciences, Beijing, 100081 People’s Republic of China

**Keywords:** Laying hen, Late phase of production, Intestinal functionality, Transcriptome, Lipid metabolism, Energy generation, Oxidation resistance

## Abstract

**Background:**

The compromised performance of laying hens in the late phase of production relative to the peak production was thought to be associated with the impairment of intestinal functionality, which plays essential roles in contributing to their overall health and production performance. In the present study, RNA sequencing was used to investigate differences in the expression profile of intestinal functionality-related genes and associated pathways between laying hens in the late phase and peak phase of production.

**Results:**

A total of 104 upregulated genes with 190 downregulated genes were identified in the ileum (the distal small intestine) of laying hens in the late phase of production compared to those at peak production. These upregulated genes were found to be enriched in little KEGG pathway, however, the downregulated genes were enriched in the pathways of PPAR signaling pathway, oxidative phosphorylation and glutathione metabolism. Besides, these downregulated genes were mapped to several GO clusters in relation to lipid metabolism, electron transport of respiratory chain, and oxidation resistance. Similarly, there were lower activities of total superoxide dismutase, glutathione S-transferase and Na^+^/K^+^-ATPase, and reductions of total antioxidant capacity and ATP level, along with an elevation in malondialdehyde content in the ileum of laying hens in the late phase of production as compared with those at peak production.

**Conclusions:**

The intestine of laying hens in the late phase of production were predominantly characterized by a disorder of lipid metabolism, concurrent with impairments of energy production and antioxidant property. This study uncovers the mechanism underlying differences between the intestinal functionality of laying hens in the late phase and peak phase of production, thereby providing potential targets for the genetic control or dietary modulation of intestinal hypofunction of laying hens in the late phase of production.

## Background

Layer industry is one of the key components contributing to sustainable food sources in the world. The late phase of production (defined as a period in which the egg production is less than 90%), accounts for a large part of the whole cycle of layer production, during which laying hens are known to be characterized by the declined production performance and poor egg quality as compared with those at peak production, resulting in a restricted economic benefit of layer production [1, 2]. One crucial reason for the compromises of production performance and egg quality of laying hens in the late phase of production could be the corresponding impairment of intestinal functional state [3, 4]. The important roles of intestinal functional state have been increasingly recognized in contributing to the overall health and production performance of poultry [5, 6], probably because the intestine possesses a wide variety of different physiological functions such as barrier function, immune defense, lipid metabolism, detoxification and neuroendocrine function [6–9], in addition to serving as the principal site for nutrient absorption. Since there was a deterioration of intestinal functioning such as absorption and barrier dysfunction, immune and defense defects in older animals as compared with young animals [10, 11], the laying hens in the late and peak phase of production were speculated to display distinct differences in terms of intestinal functioning. This could be supported by the findings that aged laying hens had a destructed intestinal structure and an increased susceptibility of gut mucosal system to lose its integrity, as well as being more vulnerable to intestinal inflammatory responses relative to the young counterparts [12, 13].

It seems that the intestinal hypofunction of laying hens in the late phase of production after having undergone the intensive metabolism at peak production is associated with the aging-related down-regulations of the expression of certain functional molecules in the intestine [14, 15], as supported by the finding that the age-related decline in the absorption of nutrients (carbohydrates, lipids and amino acids) was linked to the reduced abundances of their transporters in the intestine of rats [16, 17], besides, aging-induced disorder of energy generation in the intestine was responsible by the mitochondrial respiratory chain deficiency, being mediated by the reduced expression of cytochrome c oxidase and succinate dehydrogenase [18]. To date, comprehensive knowledge on the age-related discrepancies of intestinal functions between laying hens at different production stages is poorly understood. And far less is known regarding the differences between the intestinal functions of laying hens in the late phase and peak phase of production at the molecular level.

Digital expression profiling using next-generation sequencing promises to reduce or eliminate some weakness of microarrays. As one of the powerful next-generation sequencing techniques, RNA sequencing has expanded knowledge on the extent and complexity of transcriptomes [19]. Application of transcriptomic has been considered as an available method for nutrigenomics and physiological genomics studies in chickens, in order to obtain valuable information about the molecular mechanisms associated with the identification of key genes and pathways for the physiological changes following various treatments [20, 21]. In this study, the RNA next-generation sequencing was employed to reveal intestinal differences in transcriptome profiles of laying hens at different laying periods, aiming to identify the important genes and critical pathways associated with the underlying mechanism for differences between the complex intestinal functionality of laying hens in the late phase and peak phase of production, thereby providing potential targets for improving the performance of laying hens in the late phase of production.

## Results

### Biochemical indices of the layer intestine

The layer intestine from LP group had a reduced (*P* < 0.05) T-AOC and lower (*P* < 0.05) activities of T-SOD and GST, along with a higher (*P* < 0.05) content of MDA as compared with those from PP group (Table 1). With regard to the indices associated with energy metabolism, there were reductions (*P* < 0.05) in Na^+^/K^+^-ATPase activity and ATP level, concomitant with a decreasing trend (*P* < 0.10) of the activities of ALP and Ca^2+^/Mg^2+^-ATPase in the layer intestine of LP group relative to PP group (Table 2).

**Table 1:** Comparison of intestinal antioxidant status^1^ of laying hens between groups^2^ (*n* = 8)

	T-SOD (U/mg prot.)	GST (U/mg prot.)	T-AOC (U/mg prot.)	GSH (nmol/mg prot.)	MDA (nmol/mg prot.)
PP	65.84 ± 10.29^a^	106.78 ± 30.97^a^	11.80 ± 1.15^a^	24.91 ± 8.19	3.33 ± 0.58^b^
LP	52.99 ± 8.08^b^	77.95 ± 20.51^b^	8.49 ± 1.18^b^	20.69 ± 7.60	4.32 ± 0.74^a^
*P*-value	0.015	0.046	< 0.001	0.304	0.010

^a,b^ Values with different superscripts within the same column differ significantly (*P* < 0.05)

^*1*^ *T-SOD* total superoxide dismutase, *GST* glutathione S-transferase, *T-AOC* total antioxidant capacity, *GSH* reduced glutathione, *MDA* malondialdehyde

^*2*^ *PP* laying hens in the peak phase of production, *LP* laying hens in the late phase of production

**Table 2:** Comparison of intestinal enzyme^1^ activities of laying hens between groups^2^ (*n* = 8)

	ALP (U/mg prot.)	Na^+^/K^+^- ATPase (U/mg prot.)	Ca^2+^/Mg^2+^- ATPase (U/mg prot.)	SDH (U/mg prot.)	ATP (μmol/mg prot.)
PP	3.45 ± 0.53	1.24 ± 0.32^a^	1.19 ± 0.34	12.36 ± 4.82	0.81 ± 0.18^a^
LP	2.98 ± 0.34	0.89 ± 0.30^b^	0.92 ± 0.26	9.99 ± 3.62	0.60 ± 0.18^b^
*P*-value	0.074	0.043	0.092	0.285	0.036

^a,b^ Values with different superscripts within the same column differ significantly (*P* < 0.05)

^*1*^ *ALP* alkaline phosphatase, *SDH* succinate dehydrogenase, *ATP* adenosine triphosphate

^*2*^ *PP* laying hens in the peak phase of production, *LP* laying hens in the late phase of production

### Summary of RNA sequencing data

As shown in Table 3, RNA-Seq generated more than 40,910,976 raw reads for each library, with an average of 52,873,687 and 49,344,174 paired-end reads for the PP and LP groups, respectively. The GC contents of the libraries were ranged from 49.28 to 50.87%, which were very close to 50%. All the samples had at least 92.04% reads equal to or exceeding Q30. The majority of reads in each library were mapped to the *Gallus gallus* 5.0 assembly of the chicken genome, and the average mapping rates were 87.79 and 90.87% for PP and LP groups, respectively, which had an average of 84.32 and 87.53%, respectively, of the reads mapped to the chicken genome in an unique manner.

**Table 3:** Characteristics^1^ of RNA sequencing reads of the layer intestine (*n* = 4)

Samples^2^	GC contents (%)	Q30 (%)	Total reads	Mapped reads	Mapping ratio	Unique mapping ratio
PP1	50.67	92.88	58,014,476	52,888,432	91.16%	87.47%
PP2	50.06	92.49	50,793,752	46,281,638	91.12%	87.63%
PP 3	50.37	92.89	56,232,772	50,630,631	90.04%	86..52%
PP4	50.87	93.19	46,453,748	36,633,802	78.86%	75.66%
LP1	49.85	92.35	49,218,916	44,799,521	91.02%	87.79%
LP2	49.94	93.40	63,324,840	58,066,099	91.70%	88.36%
LP3	49.28	92.04	40,910,976	36,615,172	89.50%	86.26%
LP4	50.16	93.35	43,921,962	40,084,927	91.26%	87.70%

^*1*^*GC* guanine-cytosine, *Q30* the proportion of bases with a Phred quality score greater than 30

^*2*^*PP* laying hens in the peak phase of production, *LP* laying hens in the late phase of production

### Identification of DGEs between groups

**Fig. 1:**
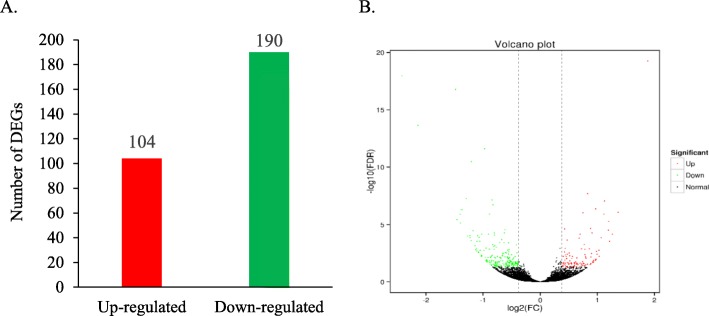
The differentially expressed genes (**a**) and their visualization by volcano plot (**b**) of the layer intestine in LP group relative to PP group (*n* = 4). LP, laying hens in the late phase of production; PP, laying hens in the peak phase of production

**Fig. 2:**
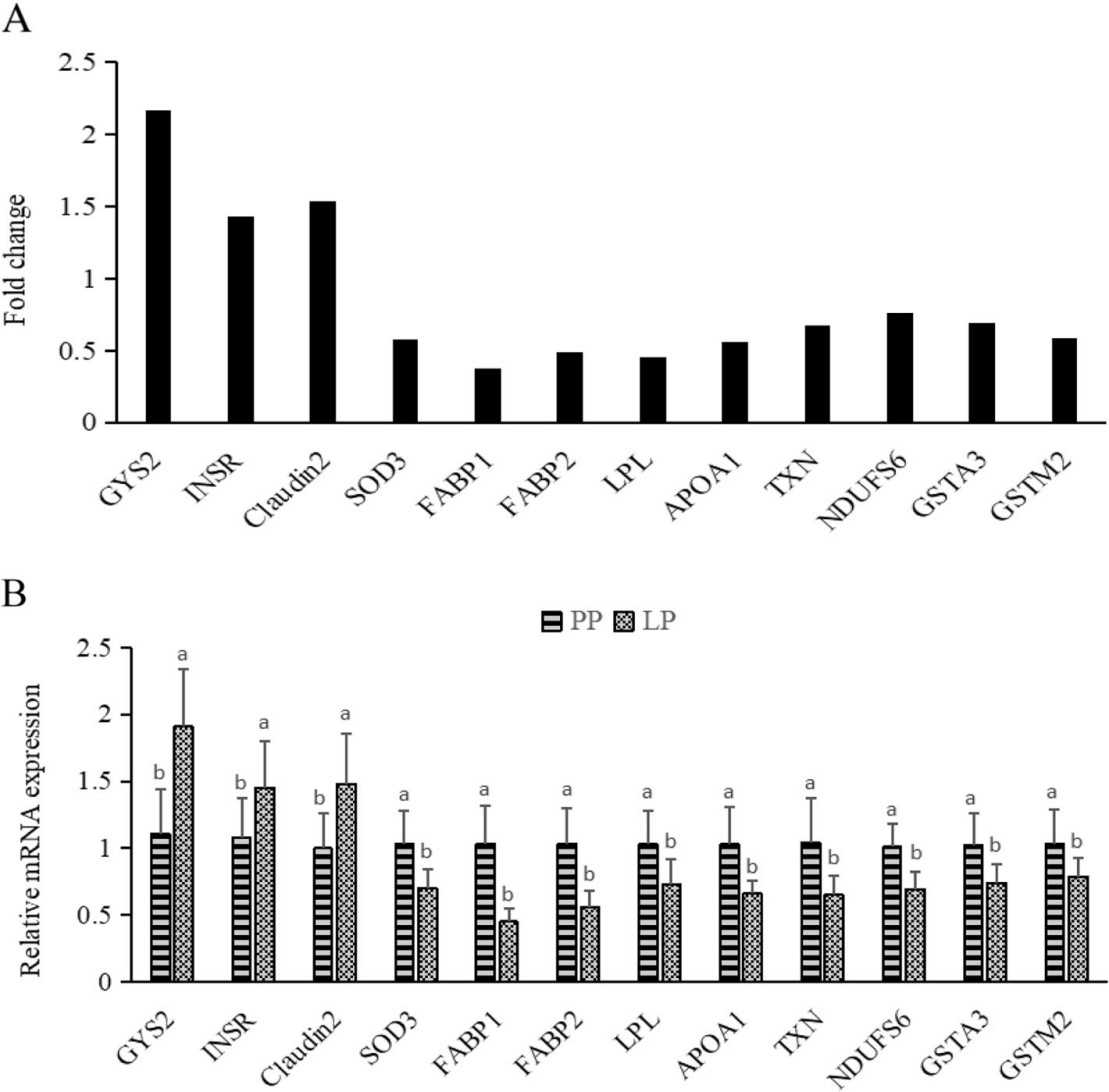
Validation of the differentially expressed genes (DEGs) by RT-PCR (*n* = 8). **a** Comparison (fold change) of the RNA-Seq data of LP group relative to PP group. **b** Individual variability of validated DGEs in RT-PCR between the PP and LP groups. LP, laying hens in the late phase of production; PP, laying hens in the peak phase of production. Values are means and standard deviations represented by vertical bars. Significance of RT-PCR data was set at *P* < 0.05, while significance of RNA-seq data was set at false discovery rate (FDR) < 0.05

There was an obvious difference in gene expression profile of the layer intestine between groups, as revealed by the principal component analysis plot (Additional file 1). A total of 294 DGEs were identified in the intestine between groups, including 104 upregulated and 190 downregulated genes in LP group relative to PP group (Fig. 1a). Volcano plot visualized the difference in the expression profile of intestinal genes in these two groups (Fig. 1b). To confirm the accuracy of RNA sequencing data, we randomly selected 12 genes including 3 upregulated genes (GYS2, INSR and Claudin-2) and 9 downregulated genes (SOD3, FABP1, FABP2, LPL, APOA1, TXN, NDUFS6, GSTM2 and GSTA3). The expression levels of these genes were quantified using RT-PCR, and the results were consistent with the findings obtained by RNA-Seq (Fig. 2), suggesting that the RNA sequencing reliably identified differentially expressed mRNAs in the ileal transcriptome.

### Functional annotation of DGEs between groups

**Fig. 3:**
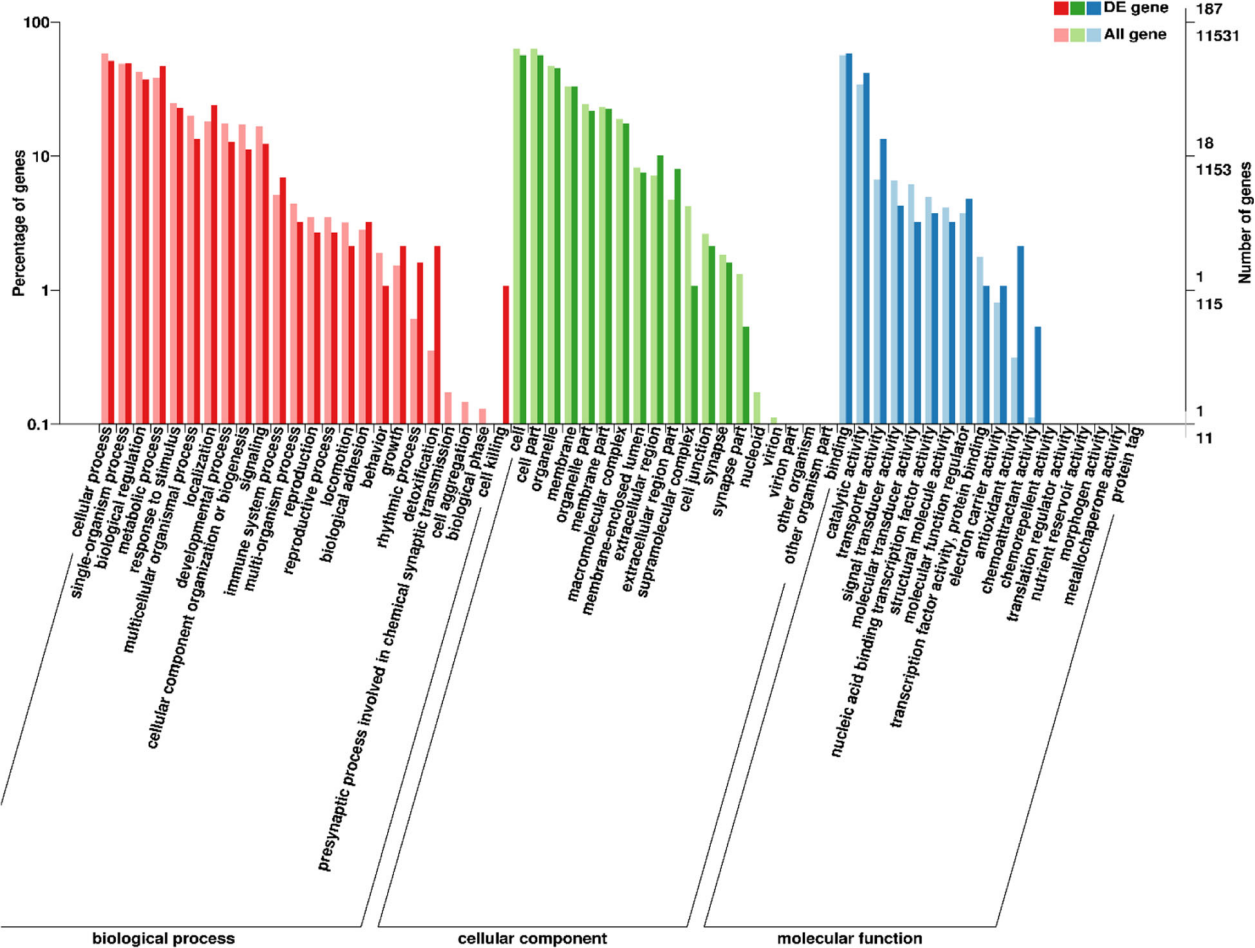
Gene oncology (GO) classification of differentially expressed genes in the layer intestine between groups (*n* = 4)

To obtain valuable information for functional prediction of DEGs, searches were made on standard unigenes in the COG and GO databases. The DEGs between groups were functionally distributed into 21 COG categories (Additional file 2). Thereinto, the greatest number of DEGs were assigned to the category of general function prediction only (25.6%), followed by the category of lipid transport and metabolism (9.6%), posttranslational modification, protein turnover, chaperones (8.8%), inorganic ion transport and metabolism (7.2%). When mapped to the GO database, the DEGs were distributed into three major functional categories including biological progress, cellular component and molecular function (Fig. 3). The most abundant terms annotated to the DEGs in the category of biological progress were cellular process, single-organism process, and metabolic process. While the most abundant terms among the category of cellular component were cell, cell part, and organelle. Within the category of molecular function, the majority of DEGs were assigned to the subcategories of binding and catalytic activity.

### Pathway enrichment analysis of DEGs between groups

The upregulated genes in LP group relative to PP group were found to confer little association (*Q* > 0.05) with any KEGG pathway except for tending to be enriched (*Q* < 0.10) in the pathway of SNARE interactions in vesicular transport (Table 4). Comparatively, the downregulated genes in LP group relative to PP group were enriched (*Q* < 0.05) in the pathways of peroxisome proliferator-activated receptor (PPAR) signaling pathway (rich factor (RF) = 11.7), oxidative phosphorylation (RF = 8.3), and glutathione metabolism (RF = 13.2) (Table 5). In addition, these downregulated genes were tended to be enriched (*Q* < 0.10) in the pathways of drug metabolism-cytochrome P450 (RF = 13.1), metabolism of xenobiotics by cytochrome P450 (RF = 12.4), and glycine, serine and threonine metabolism (RF = 11.8).

**Table 4:** Pathway analysis (top ten) of upregulated genes of the intestine of laying hens in LP group relative to PP group^1^ (*n* = 4)

Pathway name	Ko_ID	Richment _factor	*P*-value	*Q*-value
SNARE interactions in vesicular transport	ko04130	19.0	0.005	0.090
Starch and sucrose metabolism	ko00500	13.6	0.009	0.175
Cardiac muscle contraction	ko04260	9.1	0.002	0.374
Focal adhesion	ko04510	4.2	0.032	0.617
ECM-receptor interaction	ko04512	6.8	0.034	0.648
Mismatch repair	ko03430	15.1	0.064	1
Cell adhesion molecules	ko04514	4.6	0.068	1
Adrenergic signaling in cardiomyocytes	ko04261	4.5	0.071	1
Hedgehog signaling pathway	ko04340	7.4	0.127	1
Gap junction	ko04540	3.3	0.267	1

^*1*^*PP* laying hens in the peak phase of production, *LP* laying hens in the late phase of production

**Table 5:** Pathway analysis (top ten) of downregulated genes of the intestine of laying hens in LP group relative to PP group^1^ (*n* = 4)

Pathway name	Ko_ID	Richment _factor	*P*-value	*Q*-value
PPAR signaling pathway	ko03320	11.7	< 0.001	0.002
Oxidative phosphorylation	ko00190	8.3	< 0.001	0.003
Glutathione metabolism	ko00480	13.2	< 0.001	0.009
Drug metabolism - cytochrome P450	ko00982	13.1	0.001	0.059
Metabolism of xenobiotics by cytochrome P450	ko00980	12.4	0.002	0.068
Glycine, serine and threonine metabolism	ko00260	11.8	0.002	0.079
Carbon metabolism	ko01200	5.5	0.006	0.222
Glyoxylate and dicarboxylate metabolism	ko00630	10.7	0.015	0.583
Renal cell carcinoma	ko05211	53.7	0.018	0.740
Circadian rhythm	ko04710	40.3	0.025	0.984

^*1*^*PP* laying hens in the peak phase of production, *LP* laying hens in the late phase of production

In the PPAR signaling pathway, fatty acid-binding protein 1 (FABP1|FC = 0.38), FABP2 (FC = 0.49), FABP3 (FC = 0.41), FABP5 (FC = 0.69), FABP6 (FC = 0.58), lipoprotein lipase (LPL|FC = 0.56), apolipoprotein A1 (APOA1|FC = 0.56), sterol carrier protein 2 (SCP2|FC = 0.75) and perilipin-1 (PLIN1|FC = 0.59) were lower expressed in LP group relative to PP group (Table 6). While the downregulated genes in LP group that mapped to the pathway of oxidative phosphorylation were identified as following: NADH dehydrogenase (ubiquinone) Fe-S protein 6 (NDUFS6|FC = 0.76), NADH dehydrogenase (ubiquinone) 1 alpha subcomplex subunit 1 (NDUFA1|FC = 0.66), NDUFA8 (FC = 0.74), NDUFB2 (FC = 0.69), NDUFB9 (FC = 0.76), ubiquinol-cytochrome c reductase subunit 9 (UQCR9|FC = 0.65), ATP synthase subunit d (ATP5H|FC = 0.72), ATP synthase subunit e (ATP5I|FC = 0.68), ATP synthase subunit f (ATP5J|FC = 0.69), ATP synthase subunit g (ATP5L|FC = 0.66), and V-type proton ATPase subunit G 1 (ATP6V1G1|FC = 0.76). The downregulated genes in LP group that implicated in the pathway of glutathione metabolism were glutathione S-transferase (GST) omega-1 (GSTO1|FC = 0.73), GST mu 2 (GSTM2|FC = 0.59), GST alpha 3 (GSTA3|FC = 0.69) and ornithine decarboxylase 1 (ODC1|FC = 0.68). Remarkably, the downregulated expression of GSTO1, GSTM2 and GSTA3 in LP group also mediated the decreasing trend of the pathways of drug metabolism-cytochrome P450 and metabolism of xenobiotics by cytochrome P450.

**Table 6:** The differentially expressed genes^1^ (|fold change| > 1.3 at a false discovery rate < 0.05) that mapped to the enriched pathways (*n* = 4)

KEGG pathways	Pathway_ID	Differentially expressed genes (Fold change)
PPAR signaling pathway	ko03320	FABP1 (0.38), FABP2 (0.49), FABP3 (0.41), FABP5 (0.69), FABP6 (0.58), LPL (0.56), APOA1 (0.56), SCP2 (0.75), PLIN1 (0.59)
Oxidative phosphorylation	ko00190	NDUFS6 (0.76), NDUFA1 (0.66), NDUFA8 (0.74), NDUFB2 (0.69), NDUFB9 (0.76), UQCR9 (0.65), ATP5H (0.72), ATP5I (0.68), ATP5J (0.69), ATP5L (0.66), ATP6V1G1 (0.76)
Glutathione metabolism	ko00480	GSTA3 (0.69), GSTM2 (0.59), GSTO1 (0.73), ODC1 (0.68)
Drug metabolism-cytochrome P450	ko00982	GSTA3 (0.69), GSTM2 (0.59), GSTO1 (0.73)
Metabolism of xenobiotics by cytochrome P450	ko00980	GSTA3 (0.69), GSTM2 (0.59), GSTO1 (0.73)
Glycine, serine and threonine metabolism	ko00260	LOC418544 (0.55), GLDC (0.51), LOC107051323 (0.51)

^*1*^*FABP* fatty acid-binding protein, *LPL* lipoprotein lipase, *APOA* apolipoprotein A, *SCP* sterol carrier protein, *PLIN* perilipin, *NDUFS* NADH dehydrogenase (ubiquinone) Fe-S protein, *NDUFA* NADH dehydrogenase (ubiquinone) 1 alpha subcomplex subunit, *NDUFB* NADH dehydrogenase (ubiquinone) 1 beta subcomplex subunit, *UQCR* ubiquinol-cytochrome c reductase subunit, *ATP5H* ATP synthase subunit d, *ATP5I* ATP synthase subunit e, *ATP5J* ATP synthase subunit f, *ATP5L* ATP synthase subunit g, *ATP6V1G* V-type proton ATPase subunit G, *GSTA3* glutathione S-transferase alpha 3, *GSTM2* glutathione S-transferase mu 2, *GSTO1* glutathione S-transferase omega-1, *ODC1* ornithine decarboxylase 1, *LOC418544* cystathionine beta-synthase-like isoform, *GLDC* glycine dehydrogenase, *LOC107051323* glycine hydroxymethyltransferase

### GO clustering analysis of DEGs related to lipid metabolism, energy production and oxidation resistance

Since pathway analysis revealed that DEGs were predominantly enriched in the pathways of PPAR signaling pathway, oxidative phosphorylation and glutathione metabolism, the DEGs were subjected to deep-level GO clustering analysis in relation to lipid metabolism, energy generation and oxidation resistance, in order to better understand the network that responsible for the difference between groups. As shown in Table 7, there were reductions (*Q* < 0.05) of the clusters of transport, regulation of intestinal cholesterol absorption, phospholipid efflux, positive regulation of cholesterol esterification, reverse cholesterol transport, ATP synthesis coupled proton transport, hydrogen peroxide catabolic process, and removal of superoxide radicals within the category of biological process in LP group as compared to PP group. In terms of the category of cellular component, the layer intestines from LP group had less (*Q* < 0.05) clusters of very-low density lipoprotein particle and mitochondrial proton-transporting ATP synthase complex than those from PP group. Within the category of molecular function, we detected downregulated (*Q* < 0.05) clusters of lipid binding, transporter activity, phosphatidylcholine-sterol O-acyltransferase activator activity, cholesterol transporter activity, hydrogen ion transmembrane transporter activity, glutathione transferase activity, and antioxidant activity in LP group as compared with PP group.

**Table 7:** Gene oncology (GO) clustering analysis of differentially expressed genes^1^ (|fold change| > 1.3 at a false discovery rate < 0.05) in relation to lipid metabolism, energy production and oxidation resistance (*n* = 4)

GO terms	GO_ID	Differentially expressed genes (fold change)	*P*-value	*Q*-value
Biological Process				
Transport	GO:0006810	FABP6 (0.58)	0.010	0.010
Regulation of intestinal cholesterol absorption	GO:0030300	APOA1 (0.56), APOA4 (0.52)	< 0.001	0.004
ATP synthesis coupled proton transport	GO:0015986	ATP5H (0.72), ATP5I (0.68), ATP5L (0.66)	< 0.001	0.006
Phospholipid efflux	GO:0033700	APOA1 (0.56), APOA4 (0.52)	< 0.001	0.011
Positive regulation of cholesterol esterification	GO:0010873	APOA1 (0.56), APOA4 (0.52)	< 0.001	0.021
Hydrogen peroxide catabolic process	GO:0042744	PRDX1 (0.74), APOA4 (0.52)	< 0.001	0.035
Reverse cholesterol transport	GO:0043691	APOA1 (0.56), APOA4 (0.52)	< 0.001	0.035
Removal of superoxide radicals	GO:0019430	PRDX1 (0.74), APOA4 (0.52)	< 0.001	0.047
Cellular Component				
Mitochondrial proton-transporting ATP synthase complex	GO:0000276	ATP5H (0.72), ATP5I (0.68), ATP5L (0.66)	< 0.001	< 0.001
Very-low density lipoprotein particle	GO:0034361	APOA1 (0.56), APOA4 (0.52)	0.001	0.035
Molecular Function				
Lipid binding	GO:0008289	FABP1 (0.38), FABP2 (0.49), FABP3 (0.41)	0.004	0.009
Transporter activity	GO:0005215	FABP6 (0.58)	0.008	0.008
Antioxidant activity	GO:0016209	APOA4 (0.52), FABP1 (0.38)	0.001	0.008
Phosphatidylcholine-sterol O-acyltransferase activator activity	GO:0060228	APOA1 (0.56), APOA4 (0.52)	< 0.001	0.002
Glutathione transferase activity	GO:0004364	GSTA3 (0.69), GSTM2 (0.59), GSTO1 (0.73)	< 0.001	0.005
Hydrogen ion transmembrane transporter activity	GO:0015078	ATP5H (0.72), ATP5I (0.68), ATP5L (0.66)	< 0.001	0.007
Cholesterol transporter activity	GO:0017127	APOA1 (0.56), APOA4 (0.52)	< 0.001	0.027

^*1*^*FABP* fatty acid-binding protein, *APOA* apolipoprotein A, *ATP5H* ATP synthase subunit d, *ATP5I* ATP synthase subunit e, *ATP5J* ATP synthase subunit f, *ATP5L* ATP synthase subunit g, *PRDX1* peroxiredoxin-1, *GSTA3* glutathione S-transferase alpha 3, *GSTM2* glutathione S-transferase mu 2, *GSTO1* glutathione S-transferase omega-1

## Discussion

PPAR signaling pathway is a key regulator of metabolism of the intestine [22], which together with the liver are considered as important sites for lipid metabolism [7]. In the present study, the lipid metabolism-related genes such as FABP1, FABP2, FABP3, FABP5, FABP6, LPL and APOA1 that mapped to PPAR signaling pathway were downregulated in LP group relative to PP group. FABP multigene can code for diversified kinds of FABPs such as liver-type FABP (encoded by FABP1), intestinal-type FABP (encoded by FABP2), heart-type FABP (encoded by FABP3), epidermal-type FABP (encoded by FABP5), and ileal-type FABP (encoded by FABP6) [23]. These proteins display high-affinity binding for fatty acids and other hydrophobic ligands, facilitating the transport of lipids to the specific compartments of cells for storage or oxidation [24]. Although FABPs share a highly conserved structure, each of them has its own sequence and exhibits distinct affinity for ligand preferences [25]. Specifically, ileal-type FABP that located in the distal small intestine is regarded as the cytosolic receptor for bile acids, although it has a low binding affinity for fatty acids [26]. Therefore, the reduced expression of FABP6 with the resultant downregulations of GO clusters of transport and transporter activity might suggest a compromised reabsorption of luminal bile acids into enterocytes [26], resulting in a disordered regulation of lipid metabolism of the laying hens in LP group. On the other hand, the decreased expression of FABP1, FABP2 and FABP3 with the relevant downregulation of GO cluster of lipid binding were deduced to induce a malabsorption of fatty acids in LP group, since the entry of them from the lumen across the apical side of enterocytes was highly dependent on the binding by FABPs [27]. Analogously, it was indicated that the age-related decline in intestinal lipid uptake of rat is associated with a reduced abundance of FABPs [16].

The malabsorption of fatty acids in LP group could subsequently act on the nuclear receptors of PPARs, which were characterized by a DNA-binding domain and ligand-binding domains, allowing for interaction with their ligands encompassing a variety of lipid components such as fatty acids [24]. When these ligands are delivered to the nucleus under the facilitation by FABPs, the PPARs are activated and heterodimerize with retinoid receptor, thus regulating the expression of downstream target genes by binding to PPAR response elements in their promoters [28]. In this study, although no difference in the expression of PPARs was observed between groups, there might be reduced bindings of PPARs to the promoters of their downstream genes such as APOA1, LPL, FABP1, FABP3 and SCP2 in LP group [Additional file 3], leading to the corresponding reductions of these genes expression. APOA1, an essential structural and functional component of chylomicron, can be synthesized in the intestine [7]. Chylomicron can transport the absorbed triglycerides to certain parenchymal tissues such as skeletal muscle where they can release free fatty acids for oxidation under the catalysis of LPL [29], an enzyme that is nonspecifically synthesized in the intestine and spread along the vascular mesh [30]. Accordingly, the downregulations of APOA1 and LPL in LP group probably caused an inefficient utilization of dietary lipids that serve as a momentous energy source for animals, presumptively favoring the compromised performance of laying hens. Besides participating in the assembly of chylomicron, APOA1 together with APOA4 are the major functional components of very-low density lipoprotein and high density lipoprotein, being closely connected with various metabolic processes especially the cholesterol metabolism [31]. Indeed, the current study showed that the downregulated expression of APOA1 and APOA4 induced reductions of cholesterol metabolism-related GO clusters such as regulation of intestinal cholesterol absorption, cholesterol transporter activity, very-low density lipoprotein particle, positive regulation of cholesterol esterification and reverse cholesterol transport, indicating perturbations of cholesterol absorption, transport and excretion of laying hens in LP group. Phosphatidylcholine-sterol O-acyltransferase catalyzes cholesterol esterification by promoting the binding of fatty acyl group from phospholipid in high density lipoprotein to the cell-derived cholesterol [32], a process necessary for the reverse cholesterol transport. Phospholipid efflux can be conjugated with the reverse cholesterol transport from peripheral tissues to the liver, where cholesterol can be transformed into bile acids and in turn excrete to the feces [33]. Thus, the lower GO clusters of phosphatidylcholine-sterol O-acyltransferase activator activity and phospholipid efflux in LP group may exacerbate the impaired efflux of cholesterol, triggering cholesterol accumulation inside the body of laying hens in LP group.

FABP1 and FABP3 not only participate in modulation of absorption and storage of lipids, but also involved in fatty acid oxidation by promoting transport of them to mitochondria [34, 35]. SCP2 exhibits high affinity for many hydrophobic ligand**s** such as fatty acids and acyl-CoA, mediating the transport of acyl-CoA to mitochondria for oxidation [36]. Thereby, the downregulated expression of FABP1, FABP3 and SCP2 might cause an impairment of fatty acid oxidation in LP group, resulting in a lower production of substrates like NADH and FADH_2_ [37], from which the electrons could be less released and shuttled through respiratory chain. This might thus deteriorate the deficiency of oxidative phosphorylation of laying hens in LP group.

Mitochondria are the main site for oxidizing nutrients such as fatty acids to generate ATP via oxidative phosphorylation. This is accomplished by the respiratory chain in the inner mitochondrial membrane [38], comprising five complexes including complex I (NADH-CoQ dehydrogenase), complex II (succinate-CoQ dehydrogenase), complex III (reduced CoQ-cytochrome c reductase), complex IV (cytochrome C oxidase) and complex V (ATP synthase) (Additional file 4). These enzyme complexes are indispensable for the proton-coupled electron transfer during oxidative phosphorylation [37]. The gastrointestinal tract is known as an intense metabolic activity tissue with a high demand for free energy due to its roles in multiple physiological actions, accounting for as much as 15–25% of the whole energy requirement of birds [39]. Consequently, mitochondrial dysfunction could restrict nutrient absorption and metabolism, therefore favoring the declined performance of laying hens. Indeed, it was verified that feed efficiency of chickens was positively correlated with the activities of respiratory chain complexes of the intestine [40, 41]. In this study, the expression of complex I subunits (NDUFA1, NDUFA8, NDUFB2, NDUFB9 and NDUFS6), complex III subunit (UQCR9), and complex V subunits (ATP5H, ATP5I, ATP5J, ATP5L and ATP6V1G1), together with the GO clusters in association with electron transport chain coupling such as ATP synthesis coupled proton transport, mitochondrial proton-transporting ATP synthase complex, and hydrogen ion transmembrane transporter activity were all downregulated in LP group, implying a structural disorder of respiratory chain with a subsequent hypofunction of oxidative phosphorylation in LP group. Similarly, it was reported that aging induced reduced expression of the subunits of respiratory chain complexes (III, IV and V) in the brain of mice [42], as well as the subunits of all the respiratory chain complexes in rat heart [43]. We also observed that the intestine from LP group had a reduced ATP level and a lower activity of Na^+/^K^+^-ATPase, a major ion pump in basolateral membrane of enterocytes and drives the co-absorption of sodium with selected nutrients [44], confirming a disturbance of intestinal mitochondria to supply energy for laying hens in LP group. This could inevitably obstruct various metabolic processes with energy expenditure such as active transport of nutrients, presumably conducing to the impaired performance of laying hens in the late phase of production.

GSTs are encoded by GST multigene family and largely divided into groups of GST A (α), M (μ), P (π), O (ω), T (θ), D (δ), S (σ), K (κ) and Z (ζ) on the bases of biochemical and structural properties [45, 46]. GSTs are broadly spread in various cell compartments inside the body, among which GST A, M, P, K and Z can reside in the mitochondria [45]. As a crucial group of multifunctional enzymes within the body, GSTs assist with the maintenance of cellular glutathione level and play a vital role in modulating glutathione metabolism [46, Additional file 5], because they are the antioxidant enzymes with glutaredoxin-like and glutathione reductase-like activities and also associated with increased protein glutathionylation, an important modification in response to cellular redox status. These could protect respiratory chain complexes against oxidative stress [47, 48]. Specifically, GSTA3 is found to exist in the mitochondria and capable to clear various peroxidation products [45], while GSTM2 protects against mitochondrial dysfunction by acting on V-type proton ATPase [49]. GSTO1 can also be directly involved in glutathionylation of mitochondrial ATP synthase that defends against oxidative stress [50, 51]. The present study revealed that the gene expression of GSTO1, GSTM2 and GSTA3 and the activity of GST were all decreased in LP group. Similarly, the expression of GSTs in the visceral organs (liver and lung) of rats was reported to be decreased due to aging [52]. Besides, the intestine from LP group had a reduced activity of SOD, a key line of antioxidant enzyme defense systems against reactive oxygen species [53]. A decreased T-AOC coupled with an increased MDA content were also detected in LP group as compared to PP group. These findings demonstrated that the layer intestine from LP group may undergo an aggravation of oxidative stress. In support of this view, we also observed downregulations of several GO clusters related to oxidation resistance such as hydrogen peroxide catabolic process, removal of superoxide radicals, glutathione transferase activity, and antioxidant activity in LP group. Since mitochondria in the intestinal tissue is highly sensitive to oxidative stress that can lead to an inactivation of respiratory chain enzymes [54], the depressed oxidation resistance of the intestine presumably induced an inefficiency of energy production of laying hens in LP group [41]. This was in accordance with the finding that oxidative stress-induced disorder of energy production via the dysfunctional mitochondria plays a fundamental role in age-related processes [55].

In addition to involving in antioxidative activities, GSTs also represent a major cellular defense system in response to environmental hazards, as they can detoxify both endogenous and exogenous compounds such as pharmaceuticals and environmental pollutants by catalyzing the conjugation of glutathione with these compounds containing electrophilic centers, thus forming more soluble, non-toxic peptide derivatives to be excreted from the body [56]. The intestine is the primary site exposed to dietary xenobiotics that are chemical compounds foreign to the animal organism without nutritional value and considered as potential toxins [57], promoting the generation of cellular free radicals [58]. However, there were enzyme systems such as GSTs capable of biotransformation of xenobiotics in the intestine, which consequently influenced the overall bioavailability of these chemicals [56]. In this study, the reduced expression of GSTO1, GSTM2 and GSTA3 in LP group mediated a decreasing trend of pathway of metabolism of xenobiotics by cytochrome P450, being disadvantageous for detoxifying certain hazardous xenobiotics such as benzopyrene, naphthalene and aflatoxin [Additional file 6], potentially resulting in an oxidative stress in the intestine with a resultant compromise of intestinal functionality of laying hens in LP group [59].

## Conclusions

**Fig. 4:**
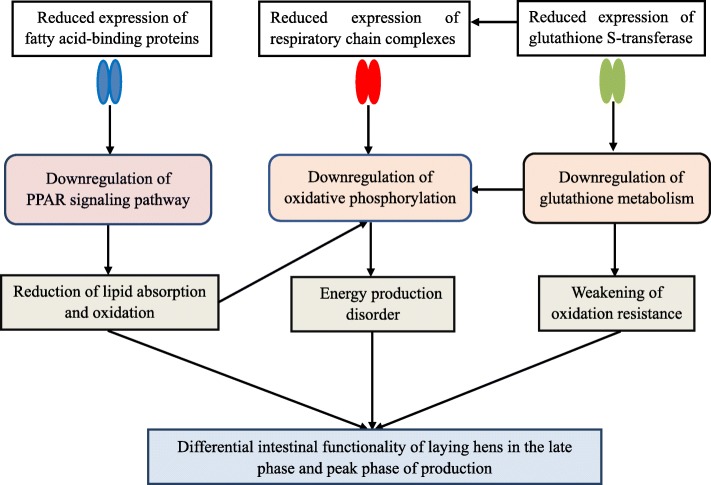
Summarization of the mechanism underlying the differential intestinal functionality of laying hens in the late phase and peak phase of production

This study demonstrated that there were disturbances of lipid metabolism, energy production and oxidation resistance of the intestine of laying hens in the late phase of production as compared to those at peak production. As summarized in Fig. 4, the impaired lipid oxidation in LP group mediated by the downregulation of PPAR signaling pathway together with the GSTs-mediated downregulation of glutathione metabolism may aggravate the dysfunction of oxidative phosphorylation, conducing to the compromised energy generation in the intestine of laying hens in the late phase of production. The results described herein provide insights into the mechanism for differences between the intestinal functionality of laying hens in the late phase and peak phase of production, which may serve as a resource for future studies on the genetic control or dietary regulation of intestinal hypofunction of laying hens in the late phase of production.

## Methods

### Animals and sample collection

The experimental animal protocol for this study was approved by the Animal Care and Use Committee of the Feed Research Institute of Chinese Academy of Agricultural Sciences. The approval number is FRI-CAAS20190527. A total of 96 Hy-Line Brown laying hens in the peak phase of production (35-wk-old, PP group) and 96 Hy-Line Brown laying hens in the late phase of production (60-wk-old, LP group) were separately allocated into 8 replicates with 12 birds per replicate cage in a randomized block design. Three birds were placed in one cage (45 × 45 × 45 cm, stocking density was 675 cm^2^/bird). The layer chicks of these two groups were obtained from Xiaoming Agriculture and Animal Husbandry Co. Ltd. (Ningxia, China) and were separately housed in two rooms with similar configurations. Three weeks before the beginning of the experiment, all the laying hens were kept in one new room to acclimate the environment and received the same diet. Besides, all birds were fed the same basal diet and allowed free access to water throughout the trial period. The composition of basal diet is shown in Additional file 7. Birds were raised in three-tier battery cages and exposed to 16 h of light/d with an intensity of 14 lx. Room temperature was maintained between 14 °C and 20 °C throughout the experiment. At the end of wk. 2 of the experiment, one bird was randomly selected from each replicate cage. The remainder laying hens were raised continuously until elimination (about 70-wk old). The selected birds were then sacrificed by cervical vertebrae dislocation and the intestinal tracts were separated. The midpoints of ileal segments were excised and put into liquid nitrogen, followed by preservation at − 80 °C for RNA extraction. Afterwards, the mucosa samples from the ileum were collected and quick-froze using liquid nitrogen, followed by storage at − 80 °C until further analysis.

### Biochemical assay of intestinal mucosa

Approximately 0.1 g of frozen mucosa sample from the ileum of laying hens from each replicate cage (*n* = 8) was homogenized with 1:10 (w/v) cold buffer (pH 7.4) containing 10 mM Tris-HCl, 0.1 mM EDTA-Na_2_ and 0.85% (w/v) NaCl. After centrifugation at 8000 rpm for 10 min at 4 °C, the resultant supernatant was collected for analysis. The adenosine triphosphatase (ATP), reduced glutathione (GSH), malondialdehyde (MDA) and total antioxidant capacity (T-AOC) were quantified colorimetrically using corresponding kits according to the manufacturer′s protocols (Jiancheng Bioengineering Institute, Nanjing, China). Meanwhile, the activities of total superoxide dismutase (T-SOD), glutathione S-transferase (GST), alkaline phosphatase (ALP), succinate dehydrogenase (SDH) and ATPase were determined using commercial kits following the manufacturer′s instructions (Jiancheng Bioengineering Institute, Nanjing, China). The results of above mentioned indices were normalized by total protein content, which was determined using a BCA protein quantitation kit (CWBiotech Co. Ltd., Beijing, China).

### RNA isolation, library preparation and sequencing

Four ileum (*n* = 4) samples per group were randomly selected for RNA isolation, which was performed by using RNeasy Mini Kit (Qiagen, Hilden, Germany) under the manufacturer′s instructions. Extracted RNA was dissolved in RNase-free water and quantified using Nanodrop 2000 spectrophotometer (Thermo Fisher Scientific, Wilmington, DE, USA). RNA integrity was evaluated by using the RNA 6000 Assay Kit at Agilent Bioanalyzer 2100 system (Agilent Technologies, Santa Clara, CA, USA). Only high-quality RNA extracts (RNA integrity number > 8) were used for library preparation.

A total of 1 μg RNA per sample was used as input material for RNA sample preparation. Four replicates from each group were analyzed independently for library synthesis and sequencing. The cDNA libraries were constructed using NEB Next Ultra RNA Library Preparation Kit following the manufacturer′s instructions (NEB Inc., Ipswich, MA, USA). Briefly, mRNA was purified from total RNA using poly-T oligo-attached magnetic beads. Fragmentation was carried out using divalent cations under elevated temperature in First Strand Synthesis Reaction Buffer (5X). The first-strand cDNA was synthesized using random hexamer primer and M-MuLV Reverse Transcriptase, followed by synthesis of the second-strand cDNA using DNA Polymerase I and RNase H. The remaining overhangs were converted into blunt ends through exonuclease/polymerase activities. After adenylation of 3′ ends of DNA fragments, the NEB Next Adaptor with hairpin loop structure were ligated to prepare for hybridization. The library fragments were purified with AMPure XP system (Beckman Coulter, Beverly, USA) in order to select cDNA fragments of preferentially 240 bp in length. The size-selected, adaptor-ligated cDNA was then incubated with 3 μL USER Enzyme (NEB Inc., Ipswich, MA, USA) at 37 °C for 15 min and 95 °C for 5 min, followed by PCR operation using Phusion High-Fidelity DNA polymerase, universal PCR primers and index (X) primer. The PCR products were purified with AMPure XP system (Beckman Coulter, Beverly, USA) and library quality was assessed using Agilent Bioanalyzer 2100 system (Agilent Technologies, CA, USA). Clustering of the index-coded samples was performed on a cBot Cluster Generation System using TruSeq PE Cluster Kit v4-cBot-HS (Illumina, San Diego, CA, USA). After cluster generation, the library preparations were sequenced via paired-end (PE150) approach on an Illumina HiSeq 2500 platform (Illumina, San Diego, USA) at Biomarker Technologies (Beijing, China). The sequencing results have been submitted to the Sequence Read Archive of the NCBI (accession number: SRR9650692).

### Transcriptomic construction

Raw reads with adapter, fuzzy N bases, rRNA, sequences shorter than 20 nt were trimmed with FasTX clipper v0.0.13, the resulting clean reads were used for the downstream analysis. Reads were mapped to the chicken reference genome (*Gallus gallus* 5.0) using TopHat v2.1.0 [60]. Mapped reads were used to estimate the gene expression level of each gene transcript. Gene function was annotated based on the following databases: Nt (NCBI non-redundant nucleotide sequences), COG (Clusters of Orthologous Groups), GO (Gene Ontology) and KO (KEGG Ortholog database). Quantification of gene expression level was estimated by fragments per kilobase of transcript per million fragments mapped (FPKM). Differential expression analysis of two conditions/groups was performed using the DESeq2, which provides statistical routines for determining differential expression in digital gene expression data using a model based on the negative binomial distribution. The resulting *P* values were adjusted using Benjamini and Hochberg′s approach for controlling the false discovery rate (FDR). The genes whose expression levels showed a |fold change, FC| > 1.3 at a FDR < 0.05 were defined as differentially expressed genes (DEGs) between groups [61, 62]. GO analysis of DEGs was implemented by the GO-seq R packages based Wallenius non-central hyper-geometric distribution [63], which can adjust for gene length bias in DEGs. Besides, KOBAS software was used to test the statistical enrichment of DEGs in KEGG pathways [64].

### Confirmation of RNA sequencing results with RT-PCR

To confirm the sequencing results, we performed quantitative RT-PCR on 12 randomly selected DGEs. Eight RNA replicates from each group were reverse transcribed to cDNA using a QuantScript RT kit with gDNA Eraser (TIANGEN Biotech. Co. Ltd., Beijing, China). RT-PCR for determining the gene expression was performed using RealMasterMix-SYBR Green kit (TIANGEN Biotech. Co. Ltd., Beijing, China) in an iCycler iQ5 multicolor real-time PCR system (Bio-Rad Laboratories, CA, USA). The β-actin was used as the housekeeping gene to normalize the amount of initial RNA of each sample. Primer sequences for β-actin and target genes are shown in Additional file 8. The protocol for gene expression was as follows: 95 °C for 5 min; 40 cycles of 95 °C for 10 s, 60 °C for 30 s. All measurements were carried out in duplicate. PCR efficiency for each gene was validated according to the slope of cDNA relative standard curve that was generated using pooled samples. Specificity of PCR products was evaluated by the analysis of melting curve. The results of relative mRNA expression of genes were calculated using the 2^-ΔΔCt^ method [65].

### Statistical analysis

Data were presented as mean with their standard deviation (SD) and analyzed by t-test procedure of the SPSS 18.0. Significance of difference of biochemical assay was defined as *P* < 0.05. While the significance regarding comparative transcriptome analysis was set at *Q* (adjusted *P* value) < 0.05, and 0.05 < *Q* < 0.10 was considered to be a tendency towards significance.

## Supplementary information

**Additional file 1:** Principal component analysis (PCA) plot of gene expression profile of the layer intestine between groups.

**Additional file 2:** Clusters of Orthologous Genes (COG) classification of differentially expressed genes of the layer intestine between groups.

**Additional file 3:** Sketch map of peroxisome proliferators-activated receptors (PPAR) signaling pathway.

**Additional file 4:** Sketch map of oxidative phosphorylation pathway.

**Additional file 5:** Sketch map of glutathione metabolism pathway.

**Additional file 6:** Sketch map of metabolism of xenobiotics by cytochrome P450.

**Additional file 7:** Composition of the basal diet.

**Additional file 8:** Sequences for real-time PCR primers.

## Data Availability

Datasets supporting the results of this article are also included in the Additional files 1-8. The RNA-seq data sets are available in the Sequence Read Archive of the NCBI (accession number: SRR9650692).

## Abbreviations

ALP: alkaline phosphatase
APOA: apolipoprotein A
ATP: adenosine triphosphatase
ATP5H: ATP synthase subunit d
ATP5I: ATP synthase subunit e
ATP5J: ATP synthase subunit f
ATP5L: ATP synthase subunit g
ATP6V1G: V-type proton ATPase subunit G
COG: Clusters of Orthologous Groups
DEGs: differentially expressed genes
FABP: fatty acid-binding protein
FC: fold change
FDR: false discovery rate
FPKM: fragments per kilobase of transcript per million fragments mapped
GO: Gene Ontology
GSH: reduced glutathione
GST: glutathione S-transferase
GSTA: glutathione S-transferase alpha
GSTM: glutathione S-transferase mu
GSTO: glutathione S-transferase omega
KO: KEGG Ortholog database
LP: laying hens in the late phase of production
LPL: lipoprotein lipase
MDA: malondialdehyde
N: NCBI non-redundant nucleotide sequences
NDUFA: NADH dehydrogenase (ubiquinone) 1 alpha subcomplex subunit
NDUFS: NADH dehydrogenase (ubiquinone) Fe-S protein
ODC: ornithine decarboxylase
PLIN: perilipin
PP: laying hens in the peak phase of production
PPAR: peroxisome proliferator-activated receptor
RF: rich factor
SCP: sterol carrier protein
SDH: succinate dehydrogenase
T-AOC: total antioxidant capacity
T-SOD: total superoxide dismutase
UQCR: ubiquinol-cytochrome c reductase subunit
