# Supplementary material for: Transcriptome analysis reveals mechanism underlying the differential intestinal functionality of laying hens in the late phase and peak phase of production
Source: BMC Genomics. 2019 Dec 12;20:970. doi: 10.1186/s12864-019-6320-y (PMC6907226; doi:10.1186/s12864-019-6320-y)
Supplement: Supplementary file 1 — Additional file 1: Principal component analysis (PCA) plot of gene expression profile of the layer intestine between groups. [file 12864_2019_6320_MOESM1_ESM.docx]

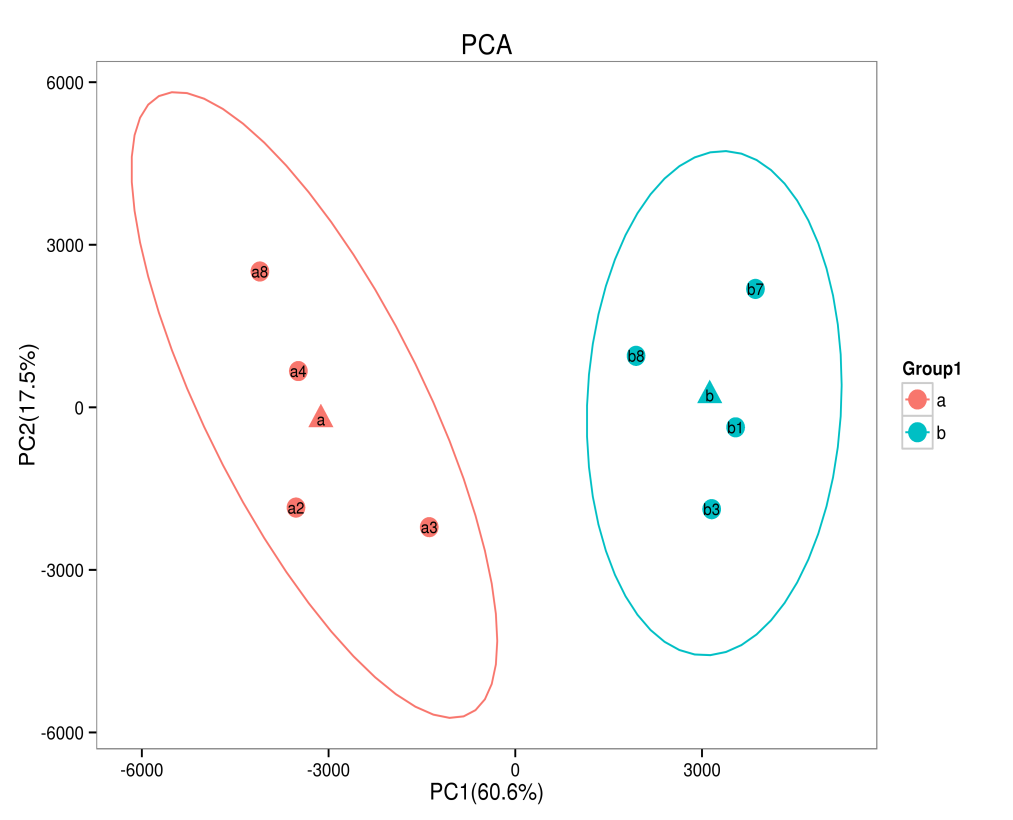


**Additional file 1** Principal component analysis (PCA) plot of gene expression profile of the layer intestine between groups.
