# Supplementary material for: Transcriptome analysis reveals mechanism underlying the differential intestinal functionality of laying hens in the late phase and peak phase of production
Source: BMC Genomics. 2019 Dec 12;20:970. doi: 10.1186/s12864-019-6320-y (PMC6907226; doi:10.1186/s12864-019-6320-y)
Supplement: Supplementary file 3 — Additional file 3: Sketch map of peroxisome proliferators-activated receptors (PPAR) signaling pathway. [file 12864_2019_6320_MOESM3_ESM.docx]

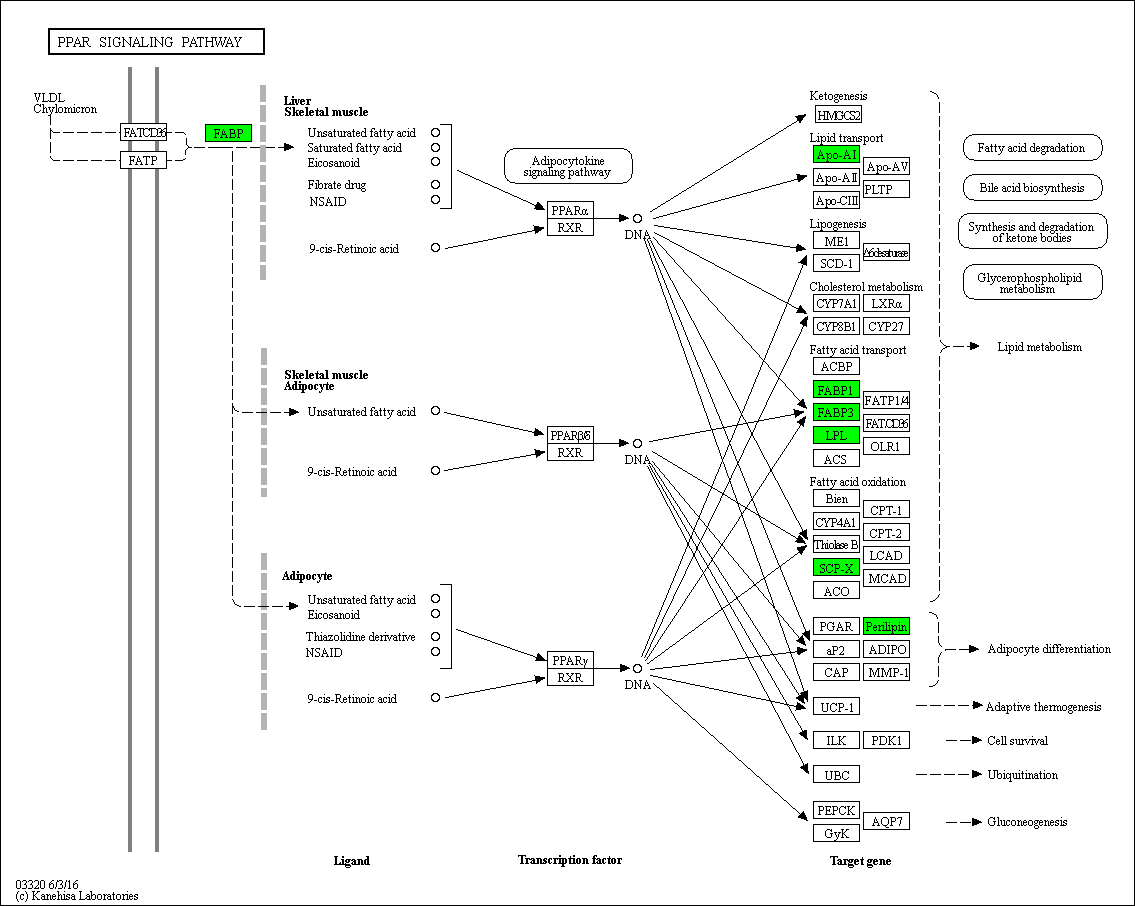


**Additional file 3** Sketch map of peroxisome proliferators-activated receptors **(**PPAR) signaling pathway.
