# Supplementary figures and images for: Transcriptome analysis reveals mechanism underlying the differential intestinal functionality of laying hens in the late phase and peak phase of production
Source: BMC Genomics. 2019 Dec 12;20:970. doi: 10.1186/s12864-019-6320-y (PMC6907226; doi:10.1186/s12864-019-6320-y)

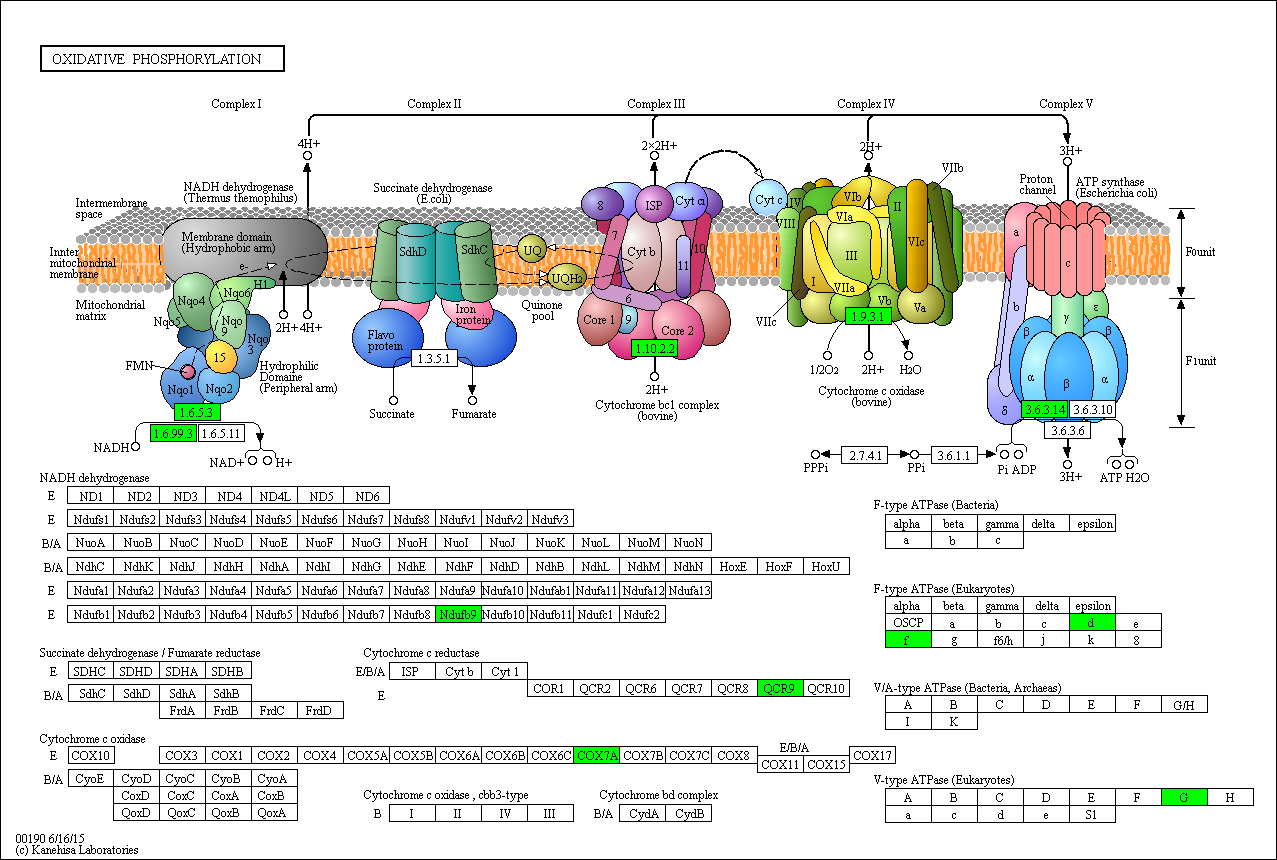


**Additional file 4** Sketch map of oxidative phosphorylation pathway.

Supplement: Supplementary file 4 — Additional file 4: Sketch map of oxidative phosphorylation pathway. [file 12864_2019_6320_MOESM4_ESM.docx]

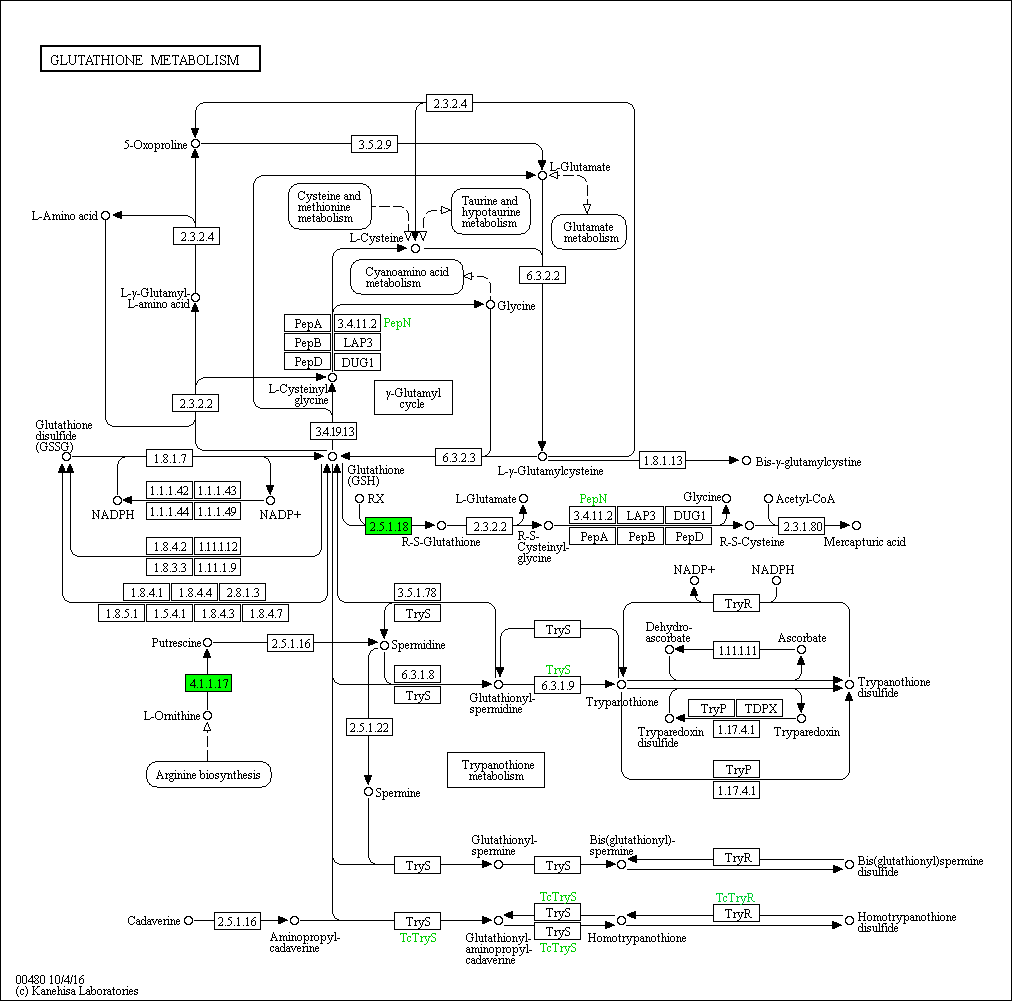


**Additional file 5** Sketch map of glutathione metabolism pathway.

Supplement: Supplementary file 5 — Additional file 5: Sketch map of glutathione metabolism pathway. [file 12864_2019_6320_MOESM5_ESM.docx]

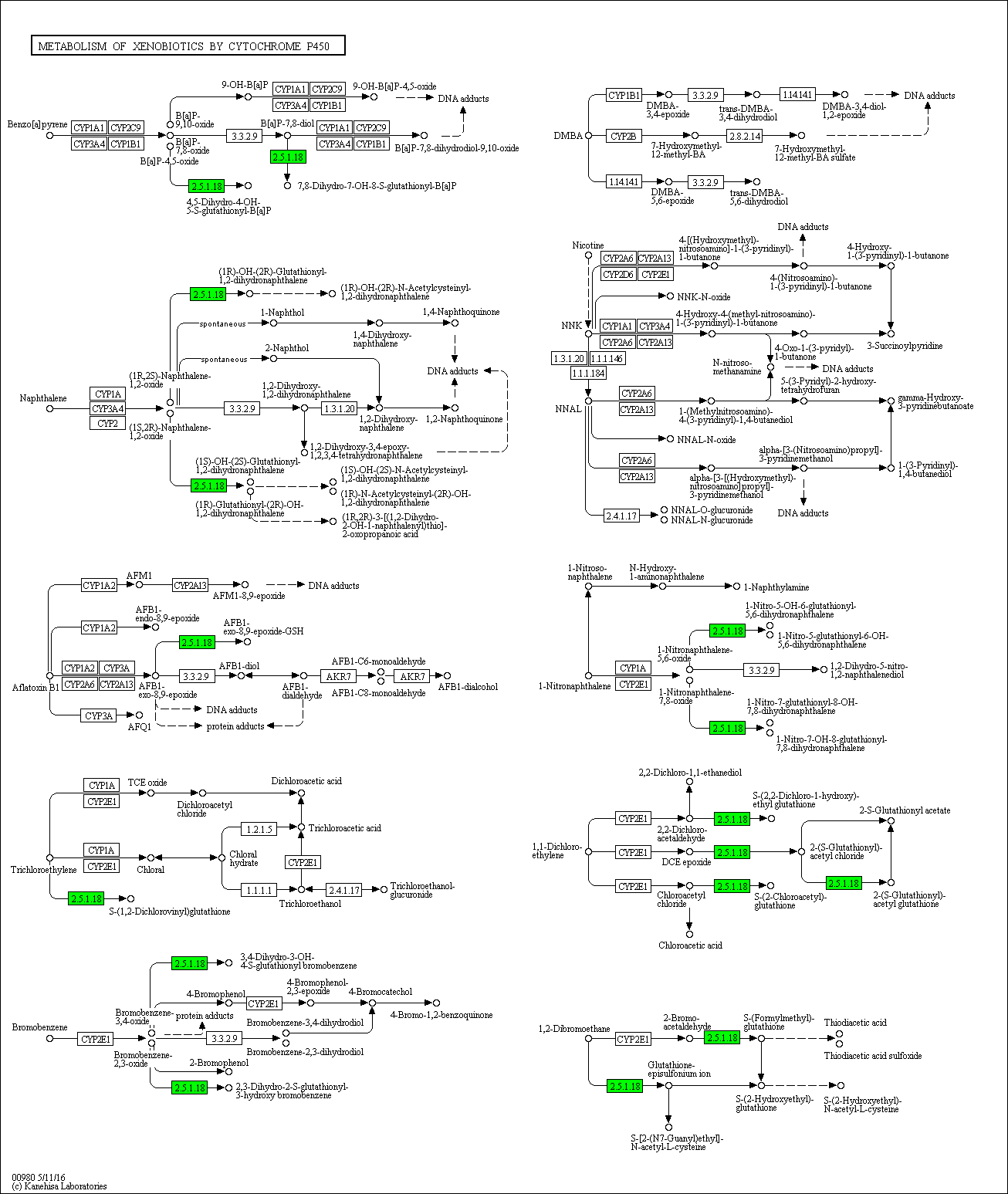


**Additional file 6** Sketch map of metabolism of xenobiotics by cytochrome P450.

Supplement: Supplementary file 6 — Additional file 6: Sketch map of metabolism of xenobiotics by cytochrome P450. [file 12864_2019_6320_MOESM6_ESM.docx]
