# Supplementary material for: Transcriptome analysis reveals mechanism underlying the differential intestinal functionality of laying hens in the late phase and peak phase of production
Source: BMC Genomics. 2019 Dec 12;20:970. doi: 10.1186/s12864-019-6320-y (PMC6907226; doi:10.1186/s12864-019-6320-y)
Supplement: Supplementary file 7 — Additional file 7: Composition of the basal diet. [file 12864_2019_6320_MOESM7_ESM.docx]

**Additional file 7** Composition of the basal diet

| Ingredients | Content, % |
| --- | --- |
| Corn | 64.52 |
| Soybean meal | 24.5 |
| Limestone | 8.9 |
| Sodium chloride | 0.3 |
| Dicalcium phosphate | 1.35 |
| Choline chloride (50%) | 0.1 |
| DL-Methionine (98%) | 0.1 |
| Multimineral^1^ | 0.2 |
| Multivitamin^2^ | 0.03 |
| Nutrient levels |  |
| Metabolizable energy (MJ/kg) | 11.18 |
| Crude protein, % | 16.16 |
| Total phosphorus, % | 0.54 |
| Available phosphorus, % | 0.34 |
| Calcium, % | 3.5 |
| Lysine, % | 0.8 |
| Methionine, % | 0.35 |
| Methionine+cysteine, % | 0.61 |

^1^ Supplied per kilogram of diet: Cu, 8 mg; Zn, 66 mg; Fe, 60 mg; Mn, 65 mg; Se, 0.3 mg; I, 1 mg.

^2^ Supplied per kilogram of diet: vitamin A, 12,500 IU; vitamin D_3_, 4,125 IU; vitamin E, 15 IU; menadione, 2 mg; thiamin, 1 mg; riboflavin, 8.5 mg; pyridoxine, 8 mg; cobalamin, 5 mg; pantothenic acid, 50 mg; niacin, 32.5 mg; biotin, 2 mg; folic acid, 5 mg; choline, 500 mg.
