# Supplementary material for: Transcriptome analysis reveals mechanism underlying the differential intestinal functionality of laying hens in the late phase and peak phase of production
Source: BMC Genomics. 2019 Dec 12;20:970. doi: 10.1186/s12864-019-6320-y (PMC6907226; doi:10.1186/s12864-019-6320-y)
Supplement: Supplementary file 8 — Additional file 8: Sequences for real-time PCR primers. [file 12864_2019_6320_MOESM8_ESM.docx]

**Additional file 8** Sequences for real-time PCR primers

| Genes^1^ | Primer sequence^2^(5′-3′) | Accession no. | References |
| --- | --- | --- | --- |
| β-Actin | F: GAGAAATTGTGCGTGACATCA | L08165 | Du et al. [1] |
|  | R: CCTGAACCTCTCATTGCCA |  |  |
| GYS2 | F: ATCGCCTTCTGTCTCTCAGC | XM_015291547.1 | Yang et al. [2] |
|  | R: TTTTGCCTATCCCTTTCAGC |  |  |
| INSR | F: CTCCCTTCTGTAACCGTGGT | XM_001233398.4 | Xie et al. [3] |
|  | R: TCTGTAGTGACGAGGATGCC |  |  |
| Claudin-2 | F: CCTGCTCACCCTCATTGGAG | NM_001277622.1 | Cheng et al. [4] |
|  | R: GCTGAACTCACTCTTGGGCT |  |  |
| SOD3 | F: TTGTGATCCATGAGCAGGAA | XM_015285700.2 | Lv et al. [5] |
|  | R: TTGTTGCAGATCCCAATCAC |  |  |
| FABP1 | F: GGGGAAGAGTGTGAGATGGA | NM_204192 | Larkina et al. [6] |
|  | R: GTTGAGTTCGGTCACGGATT |  |  |
| FABP2 | F: TGGCATTTAACGGTACTTGGA | NM_001007923 | Larkina et al. [6] |
|  | R: TCAGATTATCGTGGGCTCCT |  |  |
| LPL | F: CAGTGCAACTTCAACCATACCA | NM_205282 | Wang et al. [7] |
|  | R: AACCAGCCAGTCCACAACAA |  |  |
| APOA1 | F: GTGACCCTCGCTGTGCTCTT | NM 205525 | Jiang et al. [8] |
|  | R: CACTCAGCGTGTCCAGGTTGT |  |  |
| TXN | F: GATTTCTCTGCCACATGGTGT | NM_205453.1 | Xiao et al. [9] |
|  | R: ATCTTGGGCATCATCCACAT |  |  |
| NDUFS6 | F: ACGGCAAAAGGAGGTGAACA | XM_419061 | Brennana et al. [10] |
|  | R: CAACCTCACTCACAGGCTGC |  |  |
| GSTM2 | F: GGCAACCTGAGCCAATTCCT | NM_205090.1 | Xu et al. [11] |
|  | R: CCACCCCCCTCACTCTTTCT |  |  |
| GSTA3 | F: TTGGATAAGGCCGCAAACAGATA | NM_001001777.1 | Xu et al. [11] |
|  | R: TTTCCAGTAAATGCACGTCTGCTC |  |  |

^1^ GYS2, glycogen synthase 2; INSR, insulin receptor; SOD3, extracellular superoxide dismutase; FABP, fatty acid-binding protein; LPL, lipoprotein lipase; APOA1, apolipoprotein A1; TXN, thioredoxin; NDUFS6, NADH dehydrogenase (ubiquinone) iron-sulfur protein 6; GST, glutathione S-transferase.

^2^ F, forward; R, reverse.

**References**

[1] Du EC, Wang WW, Gan LP, Li Z, Guo SS, Guo YM. Effects of thymol and carvacrol supplementation on intestinal integrity and immune responses of broiler chickens challenged with *Clostridium perfringens*. J Anim Sci Biotechnol. 2016;7:19.

[2] Yang T, Zhao MM, Li JL, Zhang L, Jiang Y, Zhou GH, et al. In ovo feeding of creatine pyruvate alters energy metabolism in muscle of embryos and post-hatch broilers. Asian-Australas J Anim Sci. 2019;32(6):834–841.

[3] Xie Z, Zhang J, Ma S, Huang X, Huang Y. Effect of Chinese herbal medicine treatment on plasma lipid profile and hepatic lipid metabolism in Hetian broiler. Poult Sci. 2017;96(6):1918–1924.

[4] Cheng YF, Xu Q, Chen YP, Su Y, Wen C, Zhou YM. Modified palygorskite improves immunity, antioxidant ability, intestinal morphology, and barrier function in broiler chickens fed naturally contaminated diet with permitted feed concentrations of Fusarium mycotoxins. Toxins. 2018;10(11):482.

[5] Lv ZP, Fan H, Song BC, Li G, Liu D, Guo YM. Supplementing genistein for breeder hens alters the fatty acid metabolism and growth performance of offsprings by epigenetic modification. Oxid Med Cell Longev. 2019:9214209.

[6] Larkina TA, Sazanova AL, Fomichev KA, Barkova OY, Sazanov AA, Malewski T, et al. Expression profiling of candidate genes for abdominal fat mass in domestic chicken Gallus gallus. Russ J Genet. 2011;47(8):1012–1015.

[7] Wang HS, Ni XQ, Qing XD, Zeng D, Luo M, Liu L, et al. Live probiotic *Lactobacillus johnsonii* BS15 promotes growth performance and lowers fat deposition by improving lipid metabolism, intestinal development, and gut microflora in broilers. Front Microbiol. 2017;8:1073.

[8] Jiang RR, Zhao GP, Zhao JP, Chen JL, Zheng MQ, Liu RR, et al. Influence of dietary nicotinic acid supplementation on lipid metabolism and related gene expression in two distinct broiler breeds of female chickens. J Anim Physiol An N. 2014;98(5):822–829.

[9] Xiao R, Power RF, Mallonee D, Routt K, Spangler L, Pescatore AJ, et al. Effects of yeast cell wall-derived mannan-oligosaccharides on jejunal gene expression in young broiler chickens. Poultry Science. 2012;91(7):1660–1669.

[10] Brennana KM, Crowdus CA, Cantor AH, Pescatore AJ, Barger JL, Horgan K, et al. Effects of organic and inorganic dietary selenium supplementation on gene expression profiles in oviduct tissue from broiler-breeder hens. Anim Reprod Sci. 2011;125(1-4):180–188.

[11] Xu L, Zhang HJ, Yue HY, Wu SG, Yang HM, Qi GH, et al. Low-current & high-frequency electrical stunning increased oxidative stress, lipid peroxidation, and gene transcription of the mitogen-activated protein kinase/nuclear factor-erythroid 2-related factor 2/antioxidant responsive element (MAPK/Nrf2/ARE) signaling pathway in breast muscle of broilers. Food Chem. 2018;242:491–496.
